# Shape analysis of subcortical structures in obsessive‐compulsive disorder and the relationship with comorbid anxiety, depression, and medication use: A meta‐analysis by the OCD Brain Imaging Consortium

**DOI:** 10.1002/brb3.2755

**Published:** 2022-09-15

**Authors:** Jean‐Paul Fouche, Nynke A. Groenewold, Tatum Sevenoaks, Sarah Heany, Christine Lochner, Pino Alonso, Marcelo C. Batistuzzo, Narcis Cardoner, Christopher R. K. Ching, Stella J. de Wit, Boris Gutman, Marcelo Q. Hoexter, Neda Jahanshad, Minah Kim, Jun Soo Kwon, David Mataix‐Cols, Jose M. Menchon, Euripedes C. Miguel, Takashi Nakamae, Mary L. Phillips, Jesus Pujol, Yuki Sakai, Je‐Yeon Yun, Carles Soriano‐Mas, Paul M. Thompson, Kei Yamada, Dick J. Veltman, Odile A. van den Heuvel, Dan J. Stein

**Affiliations:** ^1^ Department of Psychiatry and Neuroscience Institute University of Cape Town Cape Town South Africa; ^2^ SAMRC Unit on Risk & Resilience in Mental Disorders, Department of Psychiatry Stellenbosch University Stellenbosch South Africa; ^3^ Department of Psychiatry Bellvitge University Hospital, Bellvitge Biomedical Research Institute, IDIBELL Barcelona Spain; ^4^ Carlos III Health Institute Centro de Investigacion Biomedica en Red de Salud Mental (CIBERSAM) Madrid Spain; ^5^ Department of Clinical Sciences, School of Medicine University of Barcelona Barcelona Spain; ^6^ Department & Institute of Psychiatry University of Sao Paulo Medical School Sao Paulo Brazil; ^7^ Department of Methods and Techniques in Psychology Pontifical Catholic University Sao Paulo SP Brazil; ^8^ Sant Pau Mental Health Group, Institut d'Investigacio Biomedica Sant Pau (IBB‐Sant Pau) Hospital de la Sant Creu i Sant Pau Barcelona Spain; ^9^ Department of Psychiatry and Forensic Medicine Universitat Autonoma de Barcelona Barcelona Spain; ^10^ Imaging Genetics Center USC Mark and Mary Stevens Neuroimaging and Informatics Institute, Keck School of Medicine of USC Marina del Rey California USA; ^11^ Department of Psychiatry Amsterdam UMC, Vrije Universiteit, Amsterdam Neuroscience Amsterdam The Netherlands; ^12^ Department of Anatomy & Neurosciences Amsterdam UMC, Vrije Universiteit, Amsterdam Neuroscience Amsterdam The Netherlands; ^13^ Department of Biomedical Engineering Illinois Institute of Technology Chicago Illinois USA; ^14^ Department of Neuropsychiatry Seoul National University Hospital Seoul Republic of Korea; ^15^ Department of Psychiatry Seoul National University College of Medicine Seoul Republic of Korea; ^16^ Institute of Human Behavioral Medicine SNU MRC Seoul Republic of Korea; ^17^ Department of Brain and Cognitive Sciences, College of Natural Sciences Seoul National University Seoul Republic of Korea; ^18^ Department of Clinical Neuroscience Karolinska Institute Stockholm Sweden; ^19^ Department of Psychiatry, Graduate School of Medical Science Kyoto Prefectural University of Medicine Kyoto Japan; ^20^ Department of Psychiatry University of Pittsburgh School of Medicine Pittsburgh USA; ^21^ MRI Research Unit, Radiology Department Hospital del Mar Barcelona Spain; ^22^ ATR Brain Information Communication Research Laboratory Group Kyoto Japan; ^23^ Seoul National University Hospital Seoul Republic of Korea; ^24^ Yeongeon Student Support Center Seoul National University College of Medicine Seoul Republic of Korea; ^25^ Department of Social Psychology and Quantitative Psychology Universitat de Barcelona—UB Barcelona Spain; ^26^ Department of Radiology, Graduate School of Medical Science Kyoto Prefectural University of Medicine Kyoto Japan

**Keywords:** anxiety, depression, gray matter, magnetic resonance imaging, neuroimaging, obsessive‐compulsive disorder, subcortical

## Abstract

**Objective:**

Neuroimaging studies of obsessive‐compulsive disorder (OCD) patients have highlighted the important role of deep gray matter structures. Less work has however focused on subcortical shape in OCD patients.

**Methods:**

Here we pooled brain MRI scans from 412 OCD patients and 368 controls to perform a meta‐analysis utilizing the ENIGMA‐Shape protocol. In addition, we investigated modulating effects of medication status, comorbid anxiety or depression, and disease duration on subcortical shape.

**Results:**

There was no significant difference in shape thickness or surface area between OCD patients and healthy controls. For the subgroup analyses, OCD patients with comorbid depression or anxiety had lower thickness of the hippocampus and caudate nucleus and higher thickness of the putamen and pallidum compared to controls. OCD patients with comorbid depression had lower shape surface area in the thalamus, caudate nucleus, putamen, hippocampus, and nucleus accumbens and higher shape surface area in the pallidum. OCD patients with comorbid anxiety had lower shape surface area in the putamen and the left caudate nucleus and higher shape surface area in the pallidum and the right caudate nucleus. Further, OCD patients on medication had lower shape thickness of the putamen, thalamus, and hippocampus and higher thickness of the pallidum and caudate nucleus, as well as lower shape surface area in the hippocampus and amygdala and higher surface area in the putamen, pallidum, and caudate nucleus compared to controls. There were no significant differences between OCD patients without co‐morbid anxiety and/or depression and healthy controls on shape measures. In addition, there were also no significant differences between OCD patients not using medication and healthy controls.

**Conclusions:**

The findings here are partly consistent with prior work on brain volumes in OCD, insofar as they emphasize that alterations in subcortical brain morphology are associated with comorbidity and medication status. Further work is needed to understand the biological processes contributing to subcortical shape.

## Graphical abstract

There is a paucity of literature on subcortical shape in OCD patients. In this multi‐site meta‐analysis there was no significant difference in shape between OCD patients and controls, however abnormalities in both shape thickness and surface area were found for medicated OCD patients with comorbid anxiety and depression compared to healthy controls.

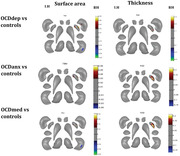

## 1 INTRODUCTION

Obsessive‐compulsive disorder (OCD) is a debilitating neuropsychiatric disorder that affects 1–3% of the population (Ruscio, Stein, Chiu & Kessler, 2010), and that is associated with significant impairment, reduced quality of life, and socio‐economic burden (Hollander, Stein, Fineberg, Marteau & Legault, 2010). Patients with OCD have been found to have functional and structural alterations in the parallel cortico‐striatal‐thalamic‐cortical circuits of the brain, as well as alterations in the fronto‐parietal, fronto‐limbic, and cerebellar regions (Milad & Rauch, 2012; van den Heuvel et al., 2016). Accordingly, there has been a focus in OCD neuroimaging research on volumetric alterations in deep gray matter structures, including the basal ganglia (Gilbert et al., 2008; Pujol et al., 2004; Szeszko et al., 2008; Zarei et al., 2011).

Reports on basal ganglia volumes in OCD have however been heterogeneous (Peng et al., 2012; Radua, van den Heuvel, Surguladze & Mataix‐Cols, 2010; Rotge et al., 2010). Prospective meta‐analyses can be useful to examine robust effects in heterogeneous populations, especially when based on collaborative and harmonized analyses of neuroimaging datasets, because this approach is less susceptible to publication bias (Radua et al., 2010; Zugman et al., 2020). Using a similar approach, a study by the ENIGMA‐OCD consortium analyzed subcortical volumes of 1,830 OCD patients and 1,759 controls and found, on average, larger pallidum, and smaller hippocampus volumes in OCD, with findings especially pronounced in patients on medication (Boedhoe et al., 2017). Age and disease duration may also impact basal ganglia findings; Pujol et al. (2004) reported that larger volumes of striatal structures in OCD were associated with older age and longer disease duration, a finding supported by the OCD Brain Imaging Consortium (OBIC) (De Wit et al., 2014) and the ENIGMA‐OCD study (Boedhoe et al., 2017). Another caveat is that many OCD patients present with comorbid anxiety and depression (Rasmussen & Eisen, 1992), and these disorders may further impact volumetric findings.

There are scarce data on shape alterations in OCD, despite the availability of several new methods for providing shape information (Shi et al., 2014; Wang et al., 2011), and growing evidence that shape variability of brain structures has an important heritable component (Roshchupkin et al., 2016). Two studies examined the shape of the hippocampus in OCD patients versus controls. The first study (Hong et al., 2007) identified surface deformities in the anterior part of the hippocampus and around the border between the body and tail, based on manual segmentation of the hippocampus. The second study (Zhang et al., 2019) showed a lateral displacement in the middle and posterior hippocampus in OCD patients using a vertex‐wise shape analysis. Moreover, one study (Zhang et al., 2019b) investigating the shape of six subcortical regions revealed right‐sided expansions in the pallidum and lateral amygdala in OCD patients using a vertex‐wise shape analysis.

The subcortical structures that have been implicated in OCD contain functionally distinct subregions. For example, the hippocampus is not a singular unit, but consists of several subregions, including the cornu ammonis subfields (CA) 1–4, dentate gyrus (DG), and the subiculum (SUB). While the more dorsal regions are involved in memory formation and spatial cognition, the ventral regions subserve affective processing (Fanselow & Dong, 2010). Similarly, the amygdala contains functionally distinct subnuclei, most importantly the basolateral and centromedial amygdala (BLA, CMA) (Mosher, Zimmerman & Gothard, 2010; Sah, Faber, Lopez de Armentia & Power, 2003). The CMA, with its reciprocal connections with the basal ganglia, midbrain, and brain stem, appears to be involved in allocating attention and generating autonomic responses to salient environmental cues. In contrast, the BLA, with its connections to the CMA and with extensive cortical regions, is primarily involved in evaluating the emotional content of sensory inputs (Mosher et al., 2010). Furthermore, the striatum is comprised of the dorsal striatum (caudate, putamen) and ventral striatum (nucleus accumbens; NAcc). The ventral putamen and nucleus accumbens have a more prominent role in emotion processing in a motivational context, whereas the dorsal putamen, along with other nuclei in the basal ganglia, is more strongly implicated in motor learning (Haber, 2016; Haber & Knutson, 2010).

To our best knowledge, no multi‐site studies have been conducted that compared the shape of subcortical regions between OCD patients and controls. The ENIGMA consortium has developed a pipeline for investigating the shape of subcortical brain regions in multi‐site studies (Gutman, Wang, Rajagopalan, Toga & Thompson, 2012; Gutman et al., 2013a, 2013b), which facilitates harmonization of brain atlases, processing methods, and statistical models across sites and meta‐analysis of the aggregated group‐level results. Notably, shape analysis allows for the exploration of subtle morphological changes with minimal manipulation of the source image (for example, without spatial smoothing). It can help to refine and extend results obtained with volumetric methods such as voxel‐based morphometry by utilizing thickness and surface area parameters that can precisely localize regional geometric shape deformations (Gutman et al., 2012, Gutman et al., 2013a, 2013b).

The present study analyzed data from OCD patients and healthy controls assessed at six academic centers across three continents (Asia, Europe, and South America). By pooling data and using standardized ENIGMA‐Shape pipelines, we aimed to have enough statistical power to compare subcortical shape between groups of OCD patients and healthy controls, as well as examine the associations of regional subcortical shape with factors such as medication status, disease duration, and psychiatric comorbidity. A meta‐analysis was performed as it can optimally account for cohort‐specific variations in sample characteristics by performing clustered analysis per cohort (Boedhoe et al., 2017). Based on previous studies (Hong et al., 2007; Zhang et al., 2019a, 2019b) and our prior mega‐analytic work in OCD (Boedhoe et al., 2017; Kong et al., 2020), we expected alterations to thickness and surface area in the pallidum, hippocampus, and amygdala in patients with OCD. For completeness, other regions of interest included the thalamus, caudate, putamen, and nucleus accumbens. Further, we expected that alterations in subcortical shape would be more pronounced in OCD patients on psychotropic medication and with longer disease duration.

## 2 METHODS

### 2.1 Participants

Data were obtained from six research centers participating in the OCD Brain Imaging Consortium OBIC. The combined cohort has been described previously (De Wit et al., 2014). More detailed information regarding demographics, comorbidity, and symptom dimensions (measured using the Yale‐Brown Obsessive‐Compulsive Scale [Y‐BOCS] (Goodman et al., 1989)) is also available in Table 1. Participants were screened for DSM‐IV axis I disorders, and exclusion criteria were ages under 18 and above 65, current psychotic disorder, recent history of a substance use disorder, intellectual disability, and severe organic or neurological pathology. Psychiatric comorbidity was allowed if OCD was the primary diagnosis. The complete sample consisted of 412 patients with primary OCD and 368 healthy controls after quality checking of data and removal of problematic scans (see De Wit et al., 2014).

**TABLE 1:** Demographic and clinical characteristics of the healthy controls (*N =* 368) and OCD patient (*N* = 412) group (as shown in De Wit et al. (2014))

Characteristic	OCD patients		Healthy controls		Statistics	
	Mean	SD	Mean	SD	*t*	*p*
Age (years) (range)	32.1 (29.4)	9.6	30.2 (28.5)	9.3	2.9	0.004
Education level (years (range)	13.7 (10.8)	2.8	14.6 (8.7)	3.1	−4.0	<0.001
YBOCS score (IQR)	24.9 (5.9)	6.2				
Age at onset of clinical symptoms (years, range)	20.1 (7.8)	8.7				
	***N***	**%**	***N***	**%**	**χ^2^**	***p***
Male	202	49.0	195	53.0	1.2	0.28
Right‐handed	354	85.9	330	89.7	1.0	0.65
**Ethnicity**					2.7	0.26
Caucasian	195	47.3	192	52.2		
Asian	171	41.5	146	39.7		
Other	6	1.5	11	3.0		
Medication use at time of scan	176	42.7	0	0.0	210.1	<0.001
Current comorbidity	149	36.2	0	0.0	210.1	<0.001
Lifetime comorbidity	213	51.7	7	1.9	253.7	<0.001
Prepubertal OCD onset	51	13.0				
**OCD symptom dimensions (YBOCS)**	**Mean**	**SD**				
Aggression/checking (IQR)	236 (13.5)	57.2				
Contamination/cleaning (IQR)	202 (28.7)	49.0				
Symmetry/ordering (IQR)	168 (34.2)	40.8				
Sexual/religious (IQR)	130 (21.5)	31.6				
Hoarding (IQR)	87 (13.6)	21.1				

### 2.2 Image processing

The 780 1.5 tesla T_1_‐weighted brain MRI scans were processed with FreeSurfer V5.3 (Fischl et al., 2002) on the Nehalem cluster at the Centre for High Performance Computing (CHPC), Rosebank, Cape Town, South Africa. This was to obtain segmentations of seven subcortical structures in both hemispheres: pallidum, hippocampus, amygdala, thalamus, caudate nucleus, putamen, and nucleus accumbens.

Segmentations were then processed using the ENIGMA shape analysis pipeline (Gutman et al., 2012, Gutman et al., 2013a, 2013b). In short, a mesh model was created for the boundary of each structure. Subcortical shapes were registered using the “Medial Demons” framework, which matches shape curvatures and medial features to a pre‐computed template (Gutman et al., 2013a, 2013b). To do this, a medial model of each individual surface model is fitted as demonstrated by Gutman et al. (2012). The medial and intrinsic features of the subcortical shape drive registration to a template parametrically on the sphere. To minimize metric distortion, the registration was performed in the fast spherical demons framework (Gutman et al., 2013a, 2013b). The templates and mean medial curves are distributed as part of the ENIGMA‐Shape package (http://enigma.usc.edu/ongoing/enigma‐shape‐analysis).

The resulting meshes for the 14 structures consisted of a total of 27,120 vertices. For these vertices, two measures were used to quantify shape, namely the radial distance and the natural logarithm of the Jacobian determinant. The radial distance represented the distance of the vertex from the medial curve of the structure and is referred to as the thickness of the structure. The Jacobian determinant captures the deformation required to map the subject‐specific vertex to a template and indicates surface dilation due to subregional volume change, offering a local measure of the surface area of the structure. The segmentations were inspected visually for any artifacts, as well as for accuracy of anatomical structure according to standardized ENIGMA protocols (http://enigma.ini.usc.edu/protocols/imaging‐protocols/)

### 2.3 Statistical analysis

For both thickness and surface area of subcortical regions, group differences between OCD patients and controls were investigated by using a general linear model (GLM) with age, sex, and intracranial volume (ICV) as covariates within each site. The resulting group‐level effect sizes and regression parameters were aggregated across sites in a random‐effects mass univariate meta‐analysis. To investigate age‐by‐diagnosis interactions, both linear and quadratic age terms were used as moderators in the main group analysis.

Next, group differences between OCD patients and controls were examined for several subsamples selected on comorbidity and medication use. OCD patients with and without lifetime depression comorbidity were each contrasted with controls, and with each other, in separate comparisons. Hereafter, OCD patients with and without lifetime anxiety comorbidity were each contrasted with controls, and with each other. Furthermore, OCD patients with and without psychotropic medication use were each contrasted with controls, and with each other. The statistical approach for these comparisons was the same as in the main analysis.

Finally, the shape correlates of clinical variability within the patient sample were assessed. Multiple regression analyses were performed to investigate the effects of OCD severity, duration of disease, age of OCD onset, and symptom dimensions (see supplementary file for detail of these covariates) within the OCD group. Sample selection was based on the availability of clinical information for each analysis. Symptom dimensions were coded as follow: 0 for lifetime absence of a dimension and 1 for lifetime presence. These dichotomous variables were then included in the multiple regression analysis.

To correct for multiple comparisons per subcortical structure, a searchlight false discovery rate (FDR) threshold (Langers, Jansen & Backes, 2007) was used at *q* = 0.05. This correction was applied locally to individual structures as we were interested in specific subcortical structures, namely the pallidum, hippocampus, and amygdala as our ROIs, in addition to other subcortical structures that were explored, namely the thalamus, caudate nucleus, putamen, and nucleus accumbens. The distance in the searchlight procedure was defined as the Euclidean distance between atlas vertices within each subcortical structure.

## 3 RESULTS

### 3.1 Sample characteristics

Patients with primary OCD (*n* = 412) and healthy controls (*n* = 368) were matched on sex, handedness, and ethnicity in individual cohorts. However, patients were significantly older (OCD group: 32.1 years [SD = 9.6]; control group: 30.2 years [SD = 9.3], *t* = 2.9, *p* = 4 × 10^−3^) and had a lower level of education (OCD group: 13.7 years [SD = 2.8]; control group: 14.6 years [SD 3.1], *t* = −4.0, *p* < 1 × 10^−3^) than the control group in the aggregated cohort. The OCD group had a mean Yale‐Brown Obsessive Compulsive Scale (Y‐BOCS) score of 24.9(SD = 6.2) and a mean age of clinical onset of 20.1 years (SD = 8.7). See De Wit et al. (2014) and Fouche et al. (2017), as well as Tables 1 and 2 for further details on the demographic and clinical characteristics of the sample.

**TABLE 2:** Demographic characteristics for OCD subgroups and healthy controls

Characteristic	OCD patients with anxiety (*n* = 83)	OCD patients without anxiety (*n* = 190)	OCD patients with depression (*n* = 101)	OCD patients without depression (*n* = 287)	OCD patients on med (*n* = 176)	OCD patients not on med (*n* = 222)	Statistics
Age (years, range) (SD)	33.8, 25.6 (8.5)	32.6, 23.9 (7.9)	31.5, 28.5 (8.2)	30.8, 29.2 (8.4)	31.2, 26.9 (6.9)	32.2, 28.1 (9.1)	*F =* 2.86; *p =* 0.89
Education level (years)	12.8, 10.1 (2.5)	13.5, 9.8 (3.1)	14.7, 10.7 (2.8)	13.2, 10.5 (2.9)	13.8, 9.9 (3.1)	14.1, 10.3 (3.0)	*F =* 3.01; *p =* 0.67
Age of onset of OCD (years, range) (SD)	19.1, 7.5 (9.2)	21.5, 8.1 (8.7)	22.1, 7.7 (9.3)	20.5, 8.2 (9.8)	21.3, 7.9 (8.9)	20.5, 8.3 (8.3)	*F =* 2.97; *p =* 0.65
Male	41 (49%)	93 (49%)	48 (48%)	140 (49%)	89 (51%)	108 (49%)	*Χ* ^2^ = 3.8; *p =* 0.87
Right‐handed	68 (82%)	168 (89%)	85 (84%)	248 (86%)	159 (90%)	195 (88%)	*χ* ^2^ = 2.9; *p =* 0.79
**Ethnicity**							
Caucasian	41 (49%)	101 (53%)	53 (52%)	132 (46%)	93 (53%)	123 (55%)	*χ* ^2^ = 2.6; *p =* 0.58
Asian	42 (51%)	87 (46%)	47 (47%)	154 (54%)	81 (46%)	99 (45%)	*χ* ^2^ = 3.7 *p* 0.89
Other	0	2 (1%)	1 (1%)	1 (< 1%)	2 (1%)	0	*χ* ^2^ = 3.2 *p* 0.79

### 3.2 Group differences in subcortical shape

#### 3.2.1 Subcortical shape in OCD patients compared to healthy controls

##### Shape surface area

There was no significant difference (*p* < 0.05 searchlight FDR‐corrected) in shape surface area for any of the subcortical structures when comparing the OCD patients (*n* = 412) to healthy controls (*n* = 368). There was also no significant age‐by‐diagnosis interaction or age^2^‐by‐diagnosis interaction for shape surface area.

##### Shape thickness

There was no significant difference (*p* < 0.05 searchlight FDR‐corrected) in shape thickness for any of the subcortical structures when comparing the OCD patients (*n* = 412) to healthy controls (*n* = 368). There was also no significant age‐by‐diagnosis interaction or age^2^‐by‐diagnosis interaction for shape thickness.

#### 3.2.2 Group differences in subcortical shape: Comorbid depressive disorder

##### Shape surface area

**FIGURE 1:**
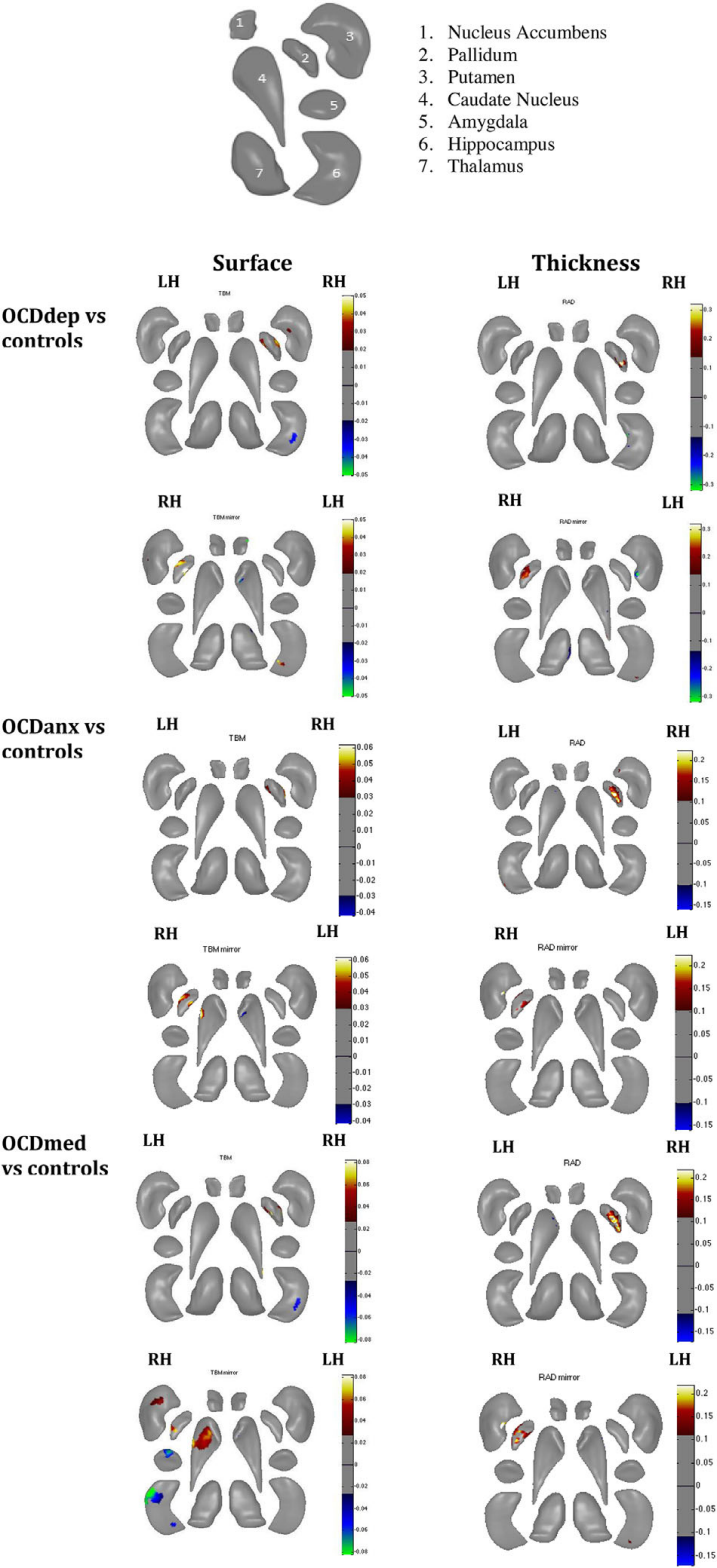
Maps showing shape differences for all the subcortical structures between OCD cohorts and controls. The legend next to each figure shows the extent of the shape change (B‐value). Results are shown at *p* < 0.05, FDR corrected. The legend at the top is a label of the different subcortical regions for clarification. RAD: Shape thickness (Radial distance); TBM: Shape surface area (Jacobian determinant); LH: left hemisphere; RH: right hemisphere; OCDmed: OCD patients on medication; OCDdep: OCD patients with major depression; OCDanx: OCD patients with comorbid anxiety.

OCD patients with comorbid depression (*n* = 101) showed lower surface area in the right hippocampus (Cohen's *d* = −0.173, 2.3% change) and higher surface area in the right pallidum (Cohen's *d* = 0.174, 4.5 % change) compared to controls (*n* = 386). Other regions that demonstrated lower surface area were the left thalamus (Cohen's *d* = −0.184, 4.7% change), left caudate nucleus (Cohen's *d* = −0.150, 7.5% change), left nucleus accumbens (Cohen's *d* = −0.162, 1.3% change) as well as right putamen (Cohen's *d* = −0.139, 6.7% change). Significant differences in shape surface area are presented in Figure 1 and Table 3.

**TABLE 3:** A summary of all the significant group comparisons of shape surface area between OCD patients and healthy controls (*p* < 0.05, corrected for multiple comparisons with FDR Searchlight)

	Shape surface area effect sizes (Cohen's *d*) and percentage change of the structure						
Group comparison	Amygdala	Hippocampus	Putamen	Caudate nucleus	Nucleus accumbens	Thalamus	Pallidum
OCDdep (*n* = 101) vs. controls (*n* = 368)	Ns	RH: *d* = −0.173 (2,3%)	RH: d = −0.139 (6.7%)	LH: *d* = −0.150 (7.5%)	LH: *d* = −0.162 (1.3%)	**LH**: d = −0.184 (4.7%)	**RH**: d = 0.174 (4.5%)
OCDanx (*n* = 190) vs. controls (*n* = 368)	Ns	Ns	RH: d = −0.212 (6.1%)	RH: *d* = 0.200 (5.8%) LH: *d* = −0.115 (4.3%)	Ns	Ns	**RH**: d = 0.178 (4.6%)
OCDmed (*n* = 176) vs. controls (*n* = 368)	RH: *d* = −0.137 (6.8%)	RH: *d* = −0.153 (5.6%)	RH: *d* = 0.132 (5.5%)	RH: *d* = 0.123 (6.2%)	Ns	Ns	RH: d = 0.144 (5.7%)

Abbreviations: Ns: not significant.;OCD: obsessive‐compulsive disorder; OCDanx: OCD patients with comorbid anxiety disorder; OCDdep: OCD patients with comorbid major depression; OCDmed: OCD patients on medication.

There was no significant difference in shape surface area between OCD patients without comorbid depression (*n* = 287) and controls (*n* = 368). There was also no significant difference between OCD patients with (*n* = 101) and without (*n* = 287) comorbid depression.

##### Shape thickness

OCD patients with comorbid depression (*n* = 101) had lower thickness in the left hippocampus (Cohen's *d* = −0.135, 5.4% change) and higher thickness in the right pallidum (Cohen's *d* = 0.159, 7.8% change) compared to controls. For the other regions, there was lower thickness in the left caudate nucleus (Cohen's *d* = −0.132, 2.8% change) and higher thickness in the right putamen (Cohen's *d* = 0.167, 6.5% change) of OCD patients when compared to controls. There was no significant difference in shape thickness or surface area between OCD patients with (*n =* 101) and without (*n =* 287) comorbid depression. Significant differences in shape thickness are presented in Figure 1 and Table 4.

**TABLE 4:** A summary of all the significant group comparisons of shape thickness between OCD patients and healthy controls (*p* < 0.05, corrected for multiple comparisons with FDR Searchlight)

	Shape thickness effect size (Cohen's *d*) and percentage change in the structure						
Group comparison	Amygdala	Hippocampus	Putamen	Caudate nucleus	Nucleus accumbens	Thalamus	Pallidum
OCDdep (*n* = 101) vs. controls (*n* = 368)	Ns	LH: *d* = −0.135 (5.4%)	RH: *d* = 0.167 (6.5%)	LH: *d* = −0.132 (2.8%)	Ns	Ns	**RH**: d = 0.159 (7.8%)
OCDanx (*n* = 190) vs. controls (*n* = 368)	Ns	LH: *d* = −0.111 (2.4%)	RH: *d* = 0.205 (7.8%)	LH: *d* = −0.139 (5.6%)	Ns	Ns	**RH**: d = 0.202 (4.3%)
OCDmed (*n* = 176) vs. controls (*n* = 368)	Ns	RH: *d* = −0.146 (4.6%)	LH: *d* = −0.115 (7.1%)	RH: *d* = 0.175 (4.4%)	Ns	**RH**: d = −0.126 (6.4%)	**RH**: d = 0.177 (5.9%)
		LH: *d* = −0.161 (5.8%)					

Abbreviations: Ns: not significant; OCD: obsessive‐compulsive disorder; OCDanx: OCD patients with comorbid anxiety disorder; OCDdep: OCD patients with comorbid major depression; OCDmed: OCD patients on medication.

There was no significant difference in shape thickness between OCD patients with comorbid depression (*n =* 287) and controls (*n =* 368), and also not between OCD patients with (*n =* 101) and without (*n =* 287) comorbid depression.

#### 3.2.3 Group differences in subcortical shape: Comorbid anxiety disorder

##### Shape surface area

OCD patients with comorbid anxiety (*n =* 83) demonstrated higher surface area in the right pallidum (Cohen's d = 0.178, 4.6% change) than controls (*n =* 368). For the other regions, there was lower surface area in the left caudate nucleus (Cohen's *d* = −0.115, 3.4% change) and right putamen (Cohen's *d* = −0.212, 6.1% change), as well as higher surface area in the right caudate nucleus (Cohen's *d* = 0.200, 5.8% change), compared to controls.

There was no significant difference in shape surface area between OCD patients without comorbid anxiety (*n =* 190) and controls (*n =* 368). There was also no significant difference between OCD patients with (*n =* 83) and without (*n =* 190) comorbid anxiety.

##### Shape thickness

OCD patients with comorbid anxiety (*n =* 83) had lower thickness in the left hippocampus (Cohen's *d* = −0.111, 2.4% change) and higher thickness in the right pallidum (Cohen's *d* = 0.202, 4.3% change) compared to controls. The exploration of other subcortical regions showed lower thickness in the left caudate nucleus (Cohen's *d* = −0.139, 5.6% change) and higher thickness in the right putamen (Cohen's *d* = 0.205, 7.8% change).

There was no significant difference in shape thickness between OCD patients without comorbid anxiety (*n =* 190) and controls (*n =* 368), and also not between OCD patients with (*n =* 83) and without (*n =* 190) comorbid anxiety.

#### 3.2.4 Group differences in subcortical shape: Psychotropic medication use

##### Shape surface area

OCD patients with medication use (*n =* 176) had lower surface area in the right hippocampus (Cohen's *d* = −0.153, 5.6% change) and right amygdala (Cohen's *d* = −0.137, 6.8% change) and higher surface area in the right pallidum (Cohen's *d* = 0.144, 5.7% change) compared to controls (*n =* 368). Other regions that demonstrated higher surface area in OCD patients with medication use were the right putamen (Cohen's *d* = 0.132, 5.5% change), and right caudate nucleus (Cohen's *d* = 0.123, 6.2% change).

There was no significant difference in shape surface area between OCD patients that did not use medication (*n =* 222) and controls (*n =* 368). There was also no significant difference between OCD patients with (*n =* 176) and without medication use (*n =* 222).

##### Shape thickness

OCD patients on psychotropic medication (*n =* 176) compared to controls (*n =* 368) had lower thickness in the bilateral hippocampus (LH: Cohen's *d* = −0.161, 5.8% change, RH: Cohen's *d* = −0.146, 4.6% change), and higher thickness in the right pallidum (Cohen's *d* = 0.177, 5.9% change) compared to controls. Other regions that demonstrated lower thickness were the left putamen (Cohen's *d* = −0.115, 7.1% change) and right thalamus (Cohen's *d* = −0.126, 6.4% change) compared to controls. There was also higher thickness of the right caudate nucleus (Cohen's *d* = 0.175, 4.4% change) in the OCD patients.

There was no significant difference in shape thickness between OCD patients without medication use (*n =* 222) and controls (*n =* 368), and also not between OCD patients with (*n =* 176) and without medication use (*n =* 222).

#### 3.2.5 Associations of other clinical variables with subcortical shape in OCD patients

Regression analyses indicated that there was no significant association of symptom dimensions (*n =* 331), total YBOCS scores (*n =* 331), duration of illness (*n =* 412), and age of onset (*n =* 412) with shape thickness or surface area for any of the subcortical regions.

## 4 DISCUSSION

Comparison of OCD patients and controls revealed no statistically significant differences in shape measures. However, alterations in shape were detected in multiple subcortical regions within OCD patients with comorbid depression and anxiety and OCD patients on medication. In line with our hypotheses, the identified alterations involved the hippocampus and pallidum for all three subgroups of OCD patients. However, the shape of the amygdala was only found to be altered in OCD patients that used medication. In addition, the caudate nucleus, putamen, and thalamus were most consistently found to have an altered shape in OCD patients with comorbidity and medication use compared to controls. Thus, this high‐powered study using high‐dimensional shape analysis identified novel shape alterations in several subcortical regions that had not been identified in previous studies with smaller samples (Hong et al., 2007; Zhang, Hu, Lu, et al., 2019; Zhang, Hu, Li, et al., 2019) in OCD patients. Moreover, age, symptom dimensions, and disease duration were not associated with shape alterations in OCD.

One of the primary regions of interest for this study was the pallidum. In OCD patients with depression or anxiety comorbidity or on medication, the external part of the pallidum consistently demonstrated higher surface area and thickness. Previous meta‐analyses have reported greater pallidum volumes in OCD (Peng et al., 2012; Radua et al., 2010; Rotge et al., 2010; Radua & Mataix‐Cols, 2009), and lower volume of this structure in anxiety disorders (Radua et al., 2010). As shape thickness and surface area are positively associated with volume (Roshchupkin et al., 2016), the findings here are consistent with this literature. One previous study in 83 OCD patients without medication use and without depression or anxiety comorbidity found that expansion of the right pallidum was associated with illness duration and symptom severity (Zhang, Hu, Li, et al., 2019). Furthermore, a smaller study in 22 OCD patients (11 with medication use and 5 with depression or anxiety comorbidity) reported trend‐level expansion of surface area in the pallidum (Shaw et al., 2015). The association observed between surface area expansion of the right pallidum and several clinical parameters could perhaps be explained by an association with global clinical severity of OCD, associated with greater comorbidity.

Other primary regions of interest included the hippocampus and amygdala. In OCD patients with depression or anxiety comorbidity, or on medication, there was also lower surface area and thickness of the hippocampal complex. The lateral ventral hippocampus has reciprocal connections with the medial prefrontal cortex and together with the amygdala is important in memory formation and emotional regulation (Izquierdo & Medina, 1997). Previous studies, including ENIGMA‐OCD studies, have reported volume abnormalities in this region in OCD (Boedhoe et al., 2017; De Wit et al., 2014; Peng et al., 2012; Radua et al., 2010; Rotge et al., 2009, 2010). It is notable that the hippocampal CA1‐3 complex is susceptible to stress‐related toxicity (Kassem et al., 2013); however, this is not necessarily specific to only OCD but can also relate to comorbid depression and anxiety. Other ENIGMA working groups focusing on major depression (Schmaal et al., 2016), schizophrenia (van Erp et al., 2016), bipolar disorder (Hibar et al., 2018), and PTSD (Logue et al., 2018) have found lower hippocampal volume in patients compared to controls.

In studies on Tourette syndrome and OCD, there is some evidence that a relative lower right amygdala volume is associated with higher scores on the aggression/checking symptom dimension (Pujol et al., 2004). Amygdala alterations have also been demonstrated in OCD and Tourette syndrome (Ludolph et al., 2008; Peterson et al., 2007; Werner et al., 2010). In addition, one pediatric study found significant amygdala volume reduction after treatment with paroxetine (Szeszko et al., 2004). The association between amygdala shape and clinical characteristics of OCD deserves further investigation in samples where more detailed clinical information is available

Other regions that were examined without hypothesis included the thalamus. In adult OCD patients on medication, there was lower thickness in the ventral lateral nucleus of the thalamus. This nucleus is responsible for relaying information from the cerebellum and pallidum to the primary motor regions, which could be associated with the motor tics seen in some patients. Although not all data are consistent (Boedhoe et al., 2017), a previous meta‐analysis that included children with OCD demonstrated a larger thalamus in the unmedicated subsample of children with OCD (Rotge et al., 2009; Rotge et al., 2010). Prior work has suggested that thalamic volume in pediatric OCD is normalized (i.e. becomes smaller) after treatment with paroxetine (Gilbert et al., 2000). A review by Piras et al. (2015) also highlighted that the thalamus is a key brain region associated with OCD in adults, with the majority of studies indicating larger volumes in this region. It is possible that thalamic enlargement is an early marker of OCD, and that medication in conjunction with brain maturation throughout adolescence acts to counteract enlargement in this region, as reflected by the smaller thickness of the ventral lateral nuclei here.

There are limited longitudinal studies that suggest that SRI treatment can normalize volume of the thalamus and putamen in OCD patients (Gilbert et al., 2000; Hoexter et al., 2012). However, from these studies, it is unclear whether medication use altered brain structure or if the changes merely reflect symptom improvement. The ENIGMA‐OCD consortium performed a large machine learning classification analysis of 2304 OCD patients and 2068 healthy controls. The analysis identified significant features that differentiated between medicated versus unmedicated OCD patients in multivariate models, including the left thalamus and pallidum (Bruin et al., 2020). These findings are in line with our shape results for the thalamus and pallidum and support the interpretation that OCD‐related alterations in brain morphometry are most pronounced in medicated patients. Future research should examine the association between subcortical shape and treatment response in OCD patients, to understand whether shape features are impacted by disease severity and can signal treatment resistance or conversely treatment responsiveness.

The putamen was another region that was investigated without hypothesis. In OCD patients with comorbid depression and anxiety, there was bilateral lower surface area and higher thickness in the putamen. Volume changes in the putamen have been associated with OCD characteristics previously (Kubota et al., 2016). Other studies have also found reduced volume and shape deformations of the bilateral putamen in patients with major depressive disorder and social anxiety disorder (Lu et al., 2016; Zhao et al., 2017). However, there are few other studies that support this finding and therefore it should be interpreted with caution. Previous VBM meta‐analyses have reported larger volumes of the putamen (Radua et al., 2010; Rotge et al., 2010) in OCD patients, both adults and children. However, there are some studies or meta‐analyses that have shown no change of the putamen and surrounding structures (Rotge et al., 2009). These discrepant VBM findings might be related to the shape changes of the putamen demonstrated here, with larger observed volume driven by putamen thickness. The simultaneous higher thickness and lower surface area may effectively mask the findings of deep gray matter volume in other studies.

Other significant findings in this study indicate lower surface area in the left nucleus accumbens in OCD patients with comorbid depression, lower surface area in the right amygdala in OCD patients on medication, and higher surface area in the right caudate nucleus as well as lower surface area in the left caudate nucleus in OCD patients with comorbid anxiety. Contrary to our other findings discussed, there is limited literature to support these findings. Future studies would benefit in investigating volume and shape changes in these subcortical regions in order to support and replicate these findings.

While patients in this study were recruited with a primary diagnosis of OCD, it is important to exclude the possibility that changes in shape of subcortical structures found here are not due to depression or anxiety comorbidity or medication use. Notably, most of the subcortical regions identified as differing in OCD are not prominently featured in studies investigating shape and volume alterations of subcortical structures in anxiety and depression (Bas‐Hoogendam et al., 2017; Schmaal et al., 2016; ; Zhao et al., 2017). Comorbidity and medication use can be markers of disease severity, and this may explain some of the consistency in findings across these relevant subsamples. In addition, the possibility that the comorbidity of OCD with depression or anxiety can possibly amplify regional brain alterations related to OCD deserves further investigation. This argues against the exclusion of these comorbidities in neuroimaging studies of OCD to allow for an exploration of the full clinical spectrum.

This study is the largest shape analysis of subcortical structures in OCD to date. Strengths of this work include (1) the large sample size, which increases the power to detect smaller statistical effects; (2) methodological homogeneity, as all data were processed and analyzed at one site and on one processing platform; (3) the high‐dimensionality of shape analysis, allowing us to investigate local differences in subcortical structures, rather than global volume; (4) in comparison to voxel based morphometry (VBM), shape analysis can distinguish between thickness and surface area; all analyses are performed in native space and abnormalities can be localized at finer scale, especially in the boundaries between structures.

Several limitations should however be emphasized. First, clinical information on comorbid depression or anxiety was not available for some of the sites; therefore, the sample selection for the relevant subanalyses differed from the whole group comparison. Second, the imaging data that were utilized here were all from 1.5T scanners, which may have lower signal‐to‐noise ratio in certain cases and thus lower sensitivity to detect group differences compared to 3T data. Third, OCD is a heterogenous disorder, and this study was not sufficiently powered to explore variations across different subtypes of OCD (Mataix‐Cols, do Rosario‐Campos & Leckman, 2005). Fourth, the use of cross‐sectional comparisons limits our ability to infer causality regarding the associations found here or to detect age‐by‐diagnosis findings. Notably, heritability estimates of shape of subcortical structures are lower in older populations compared to younger populations (Roshchupkin et al., 2016), suggesting accumulative effects of environmental factors on brain shape over time. We found no evidence that shape alterations were more pronounced in younger OCD patients, although the scope of this study was limited to adults. Future longitudinal studies may help to illuminate the neurodevelopmental and neurodegenerative aspects of alterations in subcortical shape in OCD, including changes in shape with age across the lifespan.

In summary, we found that comorbidity and medication use were associated with alterations in shape thickness and surface area in subcortical structures such as the hippocampus, pallidum, and thalamus. This is partly consistent with previous work on subcortical shape and volumes in OCD. Furthermore, our findings advance previous work by suggesting that differences in the shape of subcortical regions may occur mainly in OCD patients with comorbid depression, anxiety, and medication use. Further work is needed to delineate the exact mechanisms contributing to subcortical shape across the spectrum of OCD patients.

## CONFLICT OF INTEREST

Christopher R. K. Ching received partial grant support from Biogen, Inc. (Boston, USA) for research unrelated to this manuscript. Boris Gutman receives financial support for consulting at Natera, Inc. (San Carlos, CA, USA) in research unrelated to the manuscript. Neda Jahanshad received partial grant support from Biogen, Inc. (Boston, USA) for research unrelated to this manuscript. Paul M. Thompson received partial grant support from Biogen, Inc. (Boston, USA) for research unrelated to this manuscript. Jose M. Menchon has received research funding, consultation or lecture fees from Janssen, AbBiotics, Exeltis, and Medtronic in the last 24 months unrelated to this manuscript.

### PEER REVIEW

The peer review history for this article is available at https://publons.com/publon/10.1002/brb3.2755.

## Data Availability

The data that support the findings of this study are available on request from the corresponding author. The data are not publicly available due to privacy or ethical restrictions.
